# Evaluating the reliability of different preprocessing steps to estimate graph theoretical measures in resting state fMRI data

**DOI:** 10.3389/fnins.2015.00048

**Published:** 2015-02-19

**Authors:** Nathassia K. Aurich, José O. Alves Filho, Ana M. Marques da Silva, Alexandre R. Franco

**Affiliations:** ^1^Faculdade de Engenharia, PUCRSPorto Alegre, Brazil; ^2^Instituto do Cérebro do Rio Grande do Sul (InsCer-RS), PUCRSPorto Alegre, Brazil; ^3^Faculdade de Física, PUCRSPorto Alegre, Brazil; ^4^Faculdade de Medicina, PUCRSPorto Alegre, Brazil

**Keywords:** resting state, functional MRI, graph theory, reliability, pre-processing

## Abstract

With resting-state functional MRI (rs-fMRI) there are a variety of post-processing methods that can be used to quantify the human brain connectome. However, there is also a choice of which preprocessing steps will be used prior to calculating the functional connectivity of the brain. In this manuscript, we have tested seven different preprocessing schemes and assessed the reliability between and reproducibility within the various strategies by means of graph theoretical measures. Different preprocessing schemes were tested on a publicly available dataset, which includes rs-fMRI data of healthy controls. The brain was parcellated into 190 nodes and four graph theoretical (GT) measures were calculated; global efficiency (GEFF), characteristic path length (CPL), average clustering coefficient (ACC), and average local efficiency (ALE). Our findings indicate that results can significantly differ based on which preprocessing steps are selected. We also found dependence between motion and GT measurements in most preprocessing strategies. We conclude that by using censoring based on outliers within the functional time-series as a processing, results indicate an increase in reliability of GT measurements with a reduction of the dependency of head motion.

## Introduction

Resting-state functional MRI (rs-fMRI) is a neuroimaging method that has emerged as a powerful tool to evaluate functional connectivity patterns of the human brain (Fox and Greicius, 2010). Neuroimaging studies have demonstrated that the connectivity topology of the brain can vary depending on the mental state (Greicius, 2008), neuropsychological disorder (Bassett et al., 2008; Liu et al., 2008; Rubinov et al., 2009; Sanz-Arigita et al., 2010; Brier et al., 2014), sex (Tian et al., 2011) and even age (Achard and Bullmore, 2007; Micheloyannis et al., 2009; Wang et al., 2010; Zuo et al., 2010b, 2012). However, these studies depend on a signal (blood oxygen level dependent—BOLD) that is described by low frequency fluctuations (<0.1 Hz) (Biswal et al., 1995) and which has a very low signal to noise ratio. Thus, even the slightest source of artifacts such as physiological fluctuations (respiration and cardiac fluctuations) as well as head motion can highly influence the final estimates of connectivity. Hence, the preprocessing steps chosen to remove the fluctuations in these data caused by artifacts is extremely important (Shmueli et al., 2007; Birn, 2012).

In a recent review, Bennett and Miller (2010) highlight the importance of measuring the reliability in fMRI. They note that reliability of fMRI data is not high compared to other scientific measures, and there is still much work to be done to improve the reliability estimates. Recently, Zuo and Xing (2014) published a review addressing test-retest reliability in several functional connectivity measures. They emphasize the need to evaluate the reliability in rs-fMRI, such that, in functional connectivity measures it is important to guarantee a low variability within subjects and a high variability between subjects. The article also discusses that the choice of different preprocessing strategies can affect the reliability of rs-fMRI. For Zuo and Xing (2014), test-retest reliability “is a group-level statistic and refers to the temporal or intra-individual stability of an index measured across multiple occasions in a group of subjects.” There are a considerable amount of methods to evaluate the reliability (intraclass correlation coefficient (ICC), Pearson correlation, coefficient of variation, cluster overlap, voxel counts, etc.), and the decision depends on which post-processing method that is being evaluated. With task-based fMRI the reproducibility of the level of activation, cluster size and cluster location based on a task are of great relevance (Raemaekers et al., 2012). However, in rs-fMRI there are several choices of post-processing methodologies that evaluate the brain's functional connectivity (Margulies et al., 2010) and the reliability of each of these methods need to be measured separately.

In rs-fMRI, functional connectivity can be measured by a variety of methods including Independent Component Analysis (ICA), seed-based functional connectivity, Analysis of Low Frequency Fluctuations (ALFF), Graph Theory (GT), etc. (Margulies et al., 2010; Rubinov and Sporns, 2010). Our group has previously (Franco et al., 2013) analyzed the reliability and reproducibility of Resting State Networks (RSN), according to two post-processing methods (ICA and seed-based functional connectivity) where reliability was assessed through ICC. Results indicated that at the group level, ICA and seed based functional connectivity showed high to excellent reliability. However, at the individual level, reliability was found to be in the poor to moderate range. Using ICC, Zuo et al. (2010a) evaluated the test-test reliability of using temporally concatenated ICA (TG-ICA) combined with dual regression. They verified that there exists high test-retest reliability in rs-fMRI networks when using ICA as an analysis method. Thomason et al. (2011) studied the reliability of the connectivity map through a longitudinal study of rs-fMRI of children. Using a Pearson's correlation, they evaluated the reliability within and between sessions in six RSN. They concluded that rs-fMRI is a reliable method to assess brain networks of children. Turner et al. (2012) verified the reliability of ALFF in patients with schizophrenia. Test-rested was measured through ICC, where results indicated that the reliability to be moderate to high in healthy controls as well as chronically treated schizophrenic patients.

Reliability studies generally evaluate the test-retest of a method through the same preprocessing strategy. This is important when dealing with longitudinal studies. However, it is known that a critical source of variation between studies can arise from simply removing, including or changing parameters from the preprocessing steps on the functional data. Two independent studies, Weissenbacher et al. (2009) and Chang and Glover (2009) analyzed the influence of different pre-processing steps to evaluate functional connectivity in seed-based methods. Hallquist et al. (2013) evaluated the influence of a band-pass filter and nuisance regressors. Based on these publications, it can be observed that adding, removing or changing the order of the steps of pre-processing can substantially change the measures of functional connectivity. Moreover, Van Dijk et al. (2012), Power et al. (2013) and Satterthwaite et al. (2013) showed that head motion can introduce false correlations when estimating functional connectivity. Using the same dataset as Power et al. (2013), Jo et al. (2013) evaluated the effect of using different nuisance regression variables and evaluated the correlations between regions of interest (ROIs). This study also evaluated if censoring time points with high motion also biased their results. Jo et al. (2013) observed that by using global signal regression, the sensitivity of correlation due to motion is increased. They conclude that by despiking these data, using motion estimation regression and using a different regression parameter called a “local white matter regressor” in their preprocessing steps, it reduced the sensitivity to motion when calculating the correlation between seed pairs. Finally, they found that by using this set of nuisance variables, there is little effect on the data whether including or not the preprocessing step of censoring high motion time points.

In GT, the selection of the nodes must be carefully made, since the choice of the nodes and edges directly influence the neurobiological interpretation of the network topology (Rubinov and Sporns, 2010; Liang et al., 2012). Recently, Liang et al. (2012) evaluated three different methods of pre-processing of rs-fMRI data; with and without global signal nuisance regression, with three different band-pass filters, and with two correlation schemes for the formation of the connectivity matrix (CM). Results indicated that there is a higher reliability when calculating the CM using a Pearson's correlation vs. the use of partial correlation. Not using global signal regression as a preprocessing step also exhibited a higher reliability in comparison to including it in the preprocessing strategy. The use of different filter bands also altered the reliability of the results. Analyzing different stages of preprocessing, Braun et al. (2011) evaluated the test-retest reliability by ICC, in GT measures of 33 healthy control subjects. They found that measures of GT are dependent of preprocessing methods and parameters used in constructing the network. Before selecting the preferred preprocessing strategy, it is necessary to take into consideration that head motion can influence connectivity measures, between subjects but also between groups. Recently, Yan et al. (2013a), verified that head motion can compromise the results of test-retest reliability in several connectivity measurements. In this same year, Yan et al. (2013b) evaluated the relationship of GT measurements and head motion. With the objective of reducing the dependency of GT measurements, they applied different preprocessing methods, including a censoring based on head motion step. It was found that the preprocessing strategies tested were not sufficient to reduce dependency of GT measurements and motion estimation at the individual level, while the motion correction strategies at group level can be beneficial.

Table 1 presents different studies that used GT measures to analyze the functional connectivity in controls as well as individuals with psychological and neurological diseases. Based on this table and knowing that adding or removing different stages of preprocessing can greatly affect the final results, we propose to evaluate different preprocessing methods of rs-fMRI data and how they affect GT measures. We have chosen seven different preprocessing schemes to evaluate the reliability between them and the reproducibility within them. This has been tested on a publicly available dataset with rs-fMRI of healthy controls (http://fcon_1000.projects.nitrc.org/indi/IndiPro.html). Graph-based algorithms require the selection of network nodes. We used a parcellation mask in which the brain was segmented into 190 regions (CC200—not including the cerebellum) based on functional similarity (Craddock et al., 2012). The graph theoretical measures evaluated were global efficiency (GEFF), characteristic path length (CPL), average clustering coefficient (ACC), and average local efficiency (ALE). Through three different tests, we are evaluating which preprocessing strategy can (1) be comparable to other preprocessing strategies seen the GT rs-fMRI literature, (2) shows a low variance in connectivity measurements within a homogeneous population and finally, (3) exhibits a low dependency of GT measurement on head motion estimation.

**Table 1:** **List of preprocessing steps chosen in research articles that performed graph theoretical measurements on rs-fMRI data**.

**Study**	**Despiking**	**Band-pass filtering**	**High-pass filtering**	**Blurring**	**Motion Regression**	**WM and CSF Regression**	**Scrubbing with motion parameters**	**Polynomial Regression**	**Global Signal Regression**	**RETROICOR**	**N**
Anderson et al., 2011	x	x			x	x				x	36 controls
Cao et al., 2013		x		x	x	x					26 controls
Braun et al., 2011		x^*^		x	x	x			x		33 controls
Liu et al., 2008		x									62 (31 controls/31 patients)
Liang et al., 2012		x^**^							x^***^		47 controls
Sanz-Arigita et al., 2010			x								39 (21 controls/18 patients)
Achard et al., 2006					x						5 controls
Achard and Bullmore, 2007					x						26 (15 old/11 young controls)
Salvador et al., 2005					x						5 controls
Van den Heuvel et al., 2008		x									28 controls
Van den Heuvel et al., 2009		x									19 controls
Yan et al., 2013b		x			x^****^	x	x	x	x		158 controls

* Used two band-pass frequencies (0.04–0.08 Hz), (0.0083–0.15 Hz).

** Band-passed in three different frequency ranges: 0.01–0.1 Hz; 0.01–0.027 Hz; and 0.027–0.073 Hz.

*** Tested with and without global signal regression.

**** Performed motion regression with two different parameters; regression based on realignment: rigid-body 6-parametermodel and Friston 24-parametermode.

## Materials and methods

### Subjects

The resting state fMRI data used in this paper consists of a publicly available dataset, the 1000 Functional Connectomes Project that is part of the International Neuroimaging Data-sharing Initiative (INDI- http://fcon_1000.projects.nitrc.org/) (Biswal et al., 2010). A subset of these dataset was selected, where the inclusion criteria were; healthy control, right handed, data was collected from a homogeneous population and where there was a small variance in age between subjects. A total of 102 subject from the Peking University dataset where selected (Mean age = 11.62 ± 1.76; 43 female), which were originally used as controls for an Attention Deficit Hyperactivity Disorder (ADHD) study in children. All data were collected in a Siemens 3.0T MRI scanner with the following parameters, TR = 2000 ms, TE = 30 ms, and 240 image time points (8 min). As stated in Biswal et al. (2010), these data were approved for distribution by the institutional review boards of NYU Langone Medical Center and the New Jersey Medical School.

### Data preprocessing

Functional data were preprocessed using AFNI's “afni_proc.py” scripting algorithm (http://afni.nimh.nih.gov/pub/dist/doc/program_help/afni_proc.py.html). Seven different preprocessing strategies have been evaluated. However, for all the preprocessing strategies, these data underwent a few established preprocessing steps. Initially, the first 3 images where removed to avoid T1 effects, despiked, slice-time corrected, 3d motion corrected, nuisance regression with motion parameters, registered to MNI152 (using the T1 structural image), and scaled to percent signal change (average = 100). Registration to MNI space was visually inspected for each subject. Spatial smoothing was not performed in order to not extend blood oxygen level dependency (BOLD) signal between different regions of interest (nodes). Additionally, motion parameters, as well as the average BOLD signal of cerebrospinal fluid (CSF), white matter (WM) and whole brain where extracted for subsequent use.

The seven selected preprocessing strategies are detailed in Table 2. The different strategies are based on increasing the number of processing techniques and of what is typically seen within the resting-state literature. Bandpass filtering consists of filtering the functional data between 0.01 and 0.1 Hz. WM, CSF, and Global signal regression is a multiple regression step where the extracted CSF, WM, or Global signals are used are nuisance variables. We are evaluating two different censoring options; either censoring based on motion parameters or based on signal outliers within the BOLD data. Censoring consists of removing (censoring) time points of the functional data that pass a threshold chosen a-priori. This technique has only recently started to be used as a preprocessing step with resting state data (Power et al., 2012, 2014; Yan et al., 2013b), using motion parameters as a censoring criterion.

**Table 2:** **Description of the different preprocessing strategies evaluated**.

**Preprocessing strategy**	**Bandpass filtering**	**CSF and WM regression**	**Scrubbing with motion parameters**	**Scrubbing with outliers**	**Global signal regression**
A					
B	X				
C		X			
D	X	X			
E	X	X	X		
F	X	X		X	
G	X	X	X		X

Censoring based on motion parameters consists by initially estimating the amount of motion that occurs between subsequent images within the six motion parameters (translation: x,y,z; rotation: roll, pitch, yaw), which is calculated during the 3D motion correction preprocessing step. The amount of motion in each image is calculated by the sum of square differences in displacement:

(1) $$\begin{array}{l} Mot{\left(i\right)}_{s}=({\left(x_{i}-x_{i-1}\right)}^{2}+{\left(y_{i}-y_{i-1}\right)}^{2}+{\left(z_{i}-z_{i-1}\right)}^{2}\\ \;{+{\left(r_{i}-r_{i-1}\right)}^{2}+{\left(p_{i}-p_{i-1}\right)}^{2}+{\left(yaw_{i}-yaw_{i-1}\right)}^{2})}^{1/2} \end{array}$$

were *x, y*, and *z* are the translation estimates and *r, p, yaw* are the rotation estimates, *i* is the image, and *s* is the subject. Time points (*i*) that have motion (*Mot(i)*) above 0.2 mm were censored.

In censoring based on outliers the time points are censored based directly on the signal intensity of the voxels throughout time (Cox, 2002). Specifically, for each voxel, the median (*m(v)*0 and the median absolute deviation (*MAD(v)*) are calculated. Next, an acceptable intensity range for each voxel is defined by [*m*(*v*) − *a* · *MAD*(*v*); *m*(*v*) + *a* · *MAD*(*v*)], where *a* = *Q*^ − 1^(0.01/*N*) ^*^ (π /2)^1/2^^1^, and Q() is the reversed Gaussian cumulative distribution function (cdf) and N is the length of the time series. If a time point of a particular voxel is outside this range, it is considered an outlier. For each time point, the total amount of voxels within the brain that are outliers is calculated. Time points are censored in which 10% of voxels are considered outliers. The algorithm for estimating outliers was first presented and can be seen in detail here (Cox, 2002).

### Regions of interest (nodes)

A publically available mask was used to segment the brain into 190 regions of interest (ROIs), the CC200 (Craddock et al., 2012). The 10 regions that are contained within cerebellum were excluded from this analysis. For each subject, the average time series of the voxels within each ROI was calculated and subsequently used to calculate the graph theoretical measurements.

### Graph theory measures

The *connectivity matrix* (CM) was estimated by calculating the pair-wise correlation (Pearson's r) of the time series between each of the 190 nodes, leaving a total of 17955 correlation pairs. Before calculating the GT measures, a threshold must be applied to the CM. Four different threshold levels (absolute values) were applied to the CM (0.2, 0.3, 0.4 e 0.5) and subsequently binarized. GT measures were calculated in a freely available toolbox, Brain Connectivity Toolbox (https://sites.google.com/site/bctnet/). Global Efficiency (GEFF), Characteristic Path Length (CPL), Average Clustering Coefficient, and Average Local Efficiency (ALF) were measured for each subject, at each preprocessing strategy and threshold. A detailed review about these measures can be seen in Rubinov and Sporns (2010).

### Statistical analysis

We performed three distinct statistical analyses to evaluate the reliability and reproducibility of the different preprocessing strategies and also to assess the relationship of the GT measures with head motion estimates.

#### Test 1—reliability across different preprocessing strategies

Reliability across the seven different preprocessing strategies was evaluated. This was calculated through a repeated measures One-Way ANOVA. This statistical analysis was performed for each GT measurement and also at each threshold level (0.2, 0.3, 0.4, and 0.5), with a total of 16 One-Way ANOVAS. Follow up paired *t*-tests were also calculated in order to directly compare the different preprocessing strategies.

#### Test 2—reproducibility of the connectivity matrix

**Figure 1:**
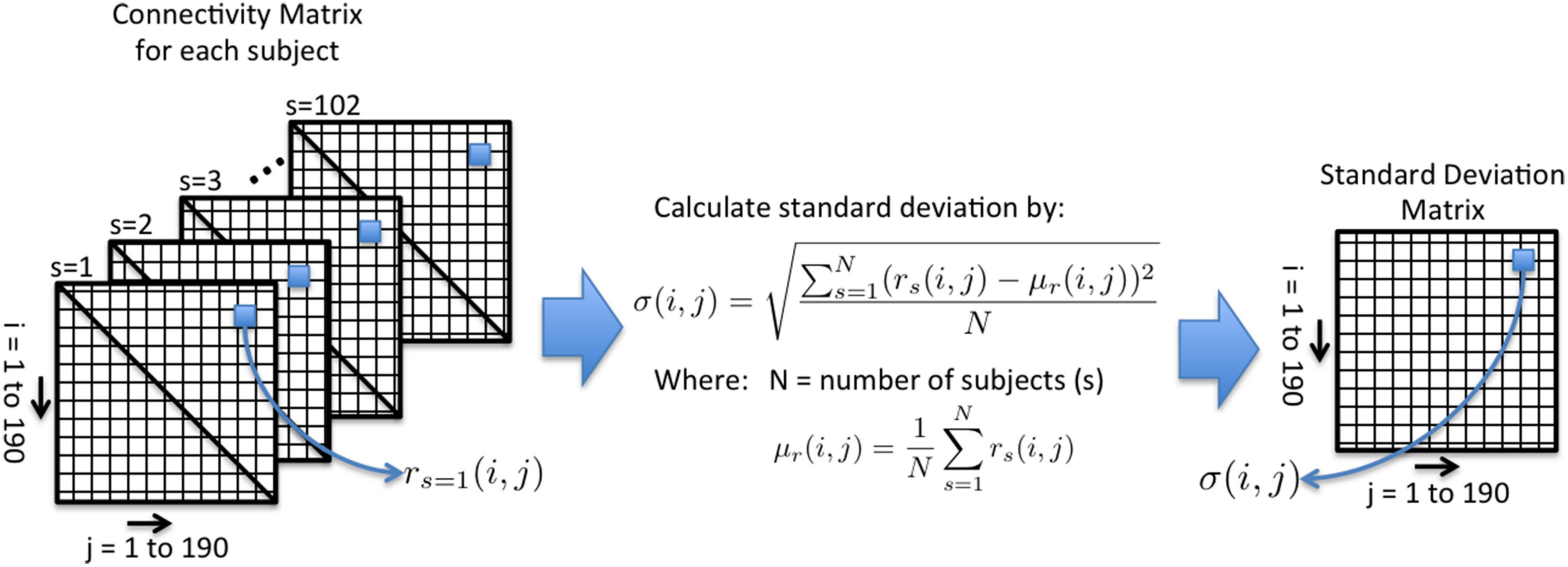
**An illustrative schematic of Test 2**. For each node pair, the standard deviation of the correlation (r_s_) is calculated across subjects(s). The Standard Deviation Matrix (SDM) is used to assess the reproducibility of a preprocessing strategy.

In order to fully evaluate how the preprocessing strategies affect the functional data, we have tested the reproducibility of the CM. The CM was evaluated directly without calculating the GT measures. For each node pair, the standard deviation was calculated across the 102 subjects. Therefore, for each preprocessing method there is a standard deviation matrix that estimates how much variance there is across subjects within each node pair. We will call these the Standard Deviation Matrices (SDM). Figure 1 illustrates a schematic of this test and the equations used to calculate the SDM. Additionally, the average correlation was calculated for each preprocessing method.

#### Test 3—motion vs. GT measures correlation

In order to evaluate these data in several quantitative measurements, we are replicating an analysis performed by Yan et al. (2013b). The relationship between the average motion and GT measurements was assessed. A correlation (Pearson's r) between each GT measurement and the average motion estimation of each subject was calculated. This test was performed to evaluate the ability to reduce or remove the dependency of GT measurements on head motion.

## Results

### Test 1—reliability across different preprocessing strategies

Mauchly's test indicated that the assumption of sphericity was violated (chi-square = 899.86, *p* < 0.001) within our data. Therefore, a repeated measure ANOVA with a Greenhouse-Geisser correction (epsilon = 0.366) was calculated. Results indicated that the mean value of all the GT measurements differed significantly [*F*_(2.194, 221.6)_ = 497.57, *p* < 0.001, partial Eta squared = 0.831] between different preprocessing strategies at all four thresholds. Since there are a total of 336 (21 pairs of preprocessing strategies, 4 levels of threshold and 4 different preprocessing methods) *post-hoc* paired *t*-tests performed (using Bonferroni correction), a summary of these tests is shown in Table 3. The grand majority of the paired *t*-tests indicated a statistical significant difference (*p* < 0.05) between the preprocessing strategies. Therefore, Table 3 only indicates the tests where the hypothesis was not rejected (*p* > 0.05), i.e., there was no statistical difference between the preprocessing strategies at a particular threshold and GT measure. Preprocessing strategies A and B differed statistically from all other preprocessing methods, consequently are not show in Table 3. Numbers indicate the total amount of paired *t*-tests that were not rejected at a particular preprocessing strategy pair and GT measure across threshold levels. For example, when comparing preprocessing strategies “C” and “G” for ACC, there was a statistical significant difference only at the 0.5 threshold level, and no difference in thresholds 0.2, 0.3, and 0.4. Hence forward the number “3” in the cell. A detailed description of all the results *post-hoc* paired *t*-tests can be seen in Supplementary Table 1.

**Table 3:** **Results from Test 1**.

**Preprocessing strategy**	**GT Measure**	**Preprocessing strategy**			
		**D**	**E**	**F**	**G**
C	GEFF				
	CPL	1		1	
	ACC	1	1	1	3
	ALE				
D	GEFF	–		4	4
	CPL	–		4	4
	ACC	–	4	4	3
	ALE	–	4	4	3
E	GEFF	–	–		
	CPL	–	–		1
	ACC	–	–	4	
	ALE	–	–	3	
F	GEFF	–	–	–	4
	CPL	–	–	–	4
	ACC	–	–	–	3
	ALE	–	–	–	4

Numbers indicate the amount of post-hoc paired t-tests (one for each threshold level) that did not show statistical differences between preprocessing strategies. Preprocessing strategies (A, B, C, …) are described in Table 2. GEFF, Global Efficiency; CPL. Characteristic Path Length; ACC, Average Cluster Coefficient; ALE, Average Local Efficiency.

### Test 2—reproducibility of the connectivity matrix

**Figure 3** exhibits the matrices that display the SDM for each preprocessing strategy and their mean value. Additionally, the average correlation score for each preprocessing strategy are; *A* = 0.2640 (±0.1301), *B* = 0.2967 (±0.1363), *C* = 0.0291 (±0.1065), *D* = 0.0251 (±0.1205), *E* = 0.0225 (±0.1218), *F* = 0.0238 (±0.1209), and *G* = 0.0009 (±0.1169). The absolute average correlation for the preprocessing strategies are; *A* = 0.2644 (±0.1293), *B* = 0.2973 (±0.1349), *C* = 0.0763 (±0.0798), *D* = 0.0859 (±0.0882), *E* = 0.0867 (±0.0885), *F* = 0.0860 (±0.0883), *G* = 0.0826 (±0.0827).

### Test 3—motion vs. GT measures correlation

Pearson's correlation scores comparing the average motion of a subject per scan and their GT measure, at each threshold, are shown in Table 4.

**Table 4:** **Correlation scores between preprocessing strategies and average motion estimation parameters**.

**GT Measurement**	**Threshold level**	**A**	**B**	**C**	**D**	**E**	**F**	**G**
GEFF	0.2	0.435	0.417	0.283	0.247	0.685	0.204	0.704
	0.3	0.442	0.412	0.319	0.247	0.691	0.218	0.703
	0.4	0.453	0.411	0.340	0.267	0.694	0.236	0.710
	0.5	0.461	0.431	0.289	0.255	0.700	0.207	0.706
	Mean (Stdev)	0.448 (±0.012)	0.418 (±0.009)	0.308 (±0.027)	0.254 (±0.009)	0.693 (±0.006)	0.216 (±0.014)	0.706 (±0.003)
CPL	0.2	–0.429	–0.414	–0.292	–0.249	–0.686	–0.207	–0.706
	0.3	–0.424	–0.413	–0.207	–0.220	–0.669	–0.200	–0.687
	0.4	–0.392	–0.388	–0.088	–0.142	–0.632	–0.132	–0.659
	0.5	–0.349	–0.376	0.056	–0.101	–0.463	–0.101	–0.454
	Mean (Stdev)	–0.399 (±0.037)	–0.398 (±0.019)	–0.133 (±0.151)	–0.178 (±0.068)	–0.613 (±0.102)	–0.160 (±0.052)	–0.627 (±0.117)
ACC	0.2	0.396	0.436	0.160	0.198	0.542	0.126	0.596
	0.3	0.424	0.455	0.282	0.203	0.407	0.103	0.455
	0.4	0.444	0.448	0.382	0.307	0.413	0.222	0.477
	0.5	0.457	0.443	0.367	0.275	0.517	0.197	0.578
	Mean (Stdev)	0.430 (±0.027)	0.446 (±0.008)	0.298 (±0.102)	0.246 (±0.054)	0.470 (±0.070)	0.162 (±0.057)	0.527 (±0.071)
ALE	0.2	0.407	0.436	0.240	0.205	0.554	0.139	0.605
	0.3	0.436	0.450	0.313	0.217	0.527	0.159	0.548
	0.4	0.444	0.421	0.364	0.282	0.549	0.222	0.591
	0.5	0.454	0.421	0.373	0.264	0.600	0.177	0.630
	Mean (Stdev)	0.435 (±0.020)	0.432 (±0.014)	0.323 (±0.061)	0.242 (±0.037)	0.558 (±0.031)	0.174 (±0.035)	0.594 (±0.034)

Threshold levels were applied to the connectivity matrix. Preprocessing strategies (A, B, C, …) are described in Table 2. GEFF, Global Efficiency; CPL, Characteristic Path Length; ACC, Average Cluster Coefficient; ALE, Average Local Efficiency; Stdev, Standard Deviation of the correlation.

### Quantity of censoring

In order to evaluate the amount of time points that where censored by each method, we calculated the quantity of time points that where censored in all 102 subjects for strategies “E” and “F.” When censoring based on motion with a threshold of 0.2 mm (Equation 1), there was an average of 6.25 (±9.75) time points removed, which represents 2.68% of the time points censored. Additionally, it was observed that 24.5% of subjects not have any time points removed. In contrast, on average only 1.07 (±4.26) of the time points were removed when censoring based on outliers (0.46% of time points), and 79.4% of the subjects did not have any time points removed.

## Discussion

The main objective of this study was to evaluate the reliability of GT measurements across different preprocessing strategies and also test the reproducibility within a particular strategy. For us to be able to apply fMRI, or more specifically, rs-fMRI within a clinical setting, we must first critically evaluate its reliability and reproducibility at the subject level (Bennett and Miller, 2010). There are many sources of variability in fMRI data and unfortunately the steps chosen in the preprocessing pipeline can be one of these variables. Based on Table 1, we can see that there isn't a consensus on which preprocessing strategy is the best to be applied to rs-fMRI data. Therefore, if there isn't even a consensus on the preprocessing strategies used to preprocess functional data, how can we establish if fMRI is reliable or not? We have chosen to test some of the mostly used preprocessing strategies for resting state data (Table 2), including a novel method, censoring by two different quantitative measurements (motion and BOLD signal outliers).

With a large (*N* = 102) homogeneous group, we evaluated whether seven different preprocessing strategies can significantly change GT measurements. Analyzing results from Table 3, final measurements can be altered significantly simply by including or removing a preprocessing step. Only preprocessing strategies “D” and “F” do not differ in any of the different threshold levels and GT measurements tested. The difference between pipelines “D” and “F,” is that “F” includes a censoring based on outliers step. Also based on Table 3, preprocessing strategy G has similar results to D and F is almost every GT measurement.

**Figure 2:**
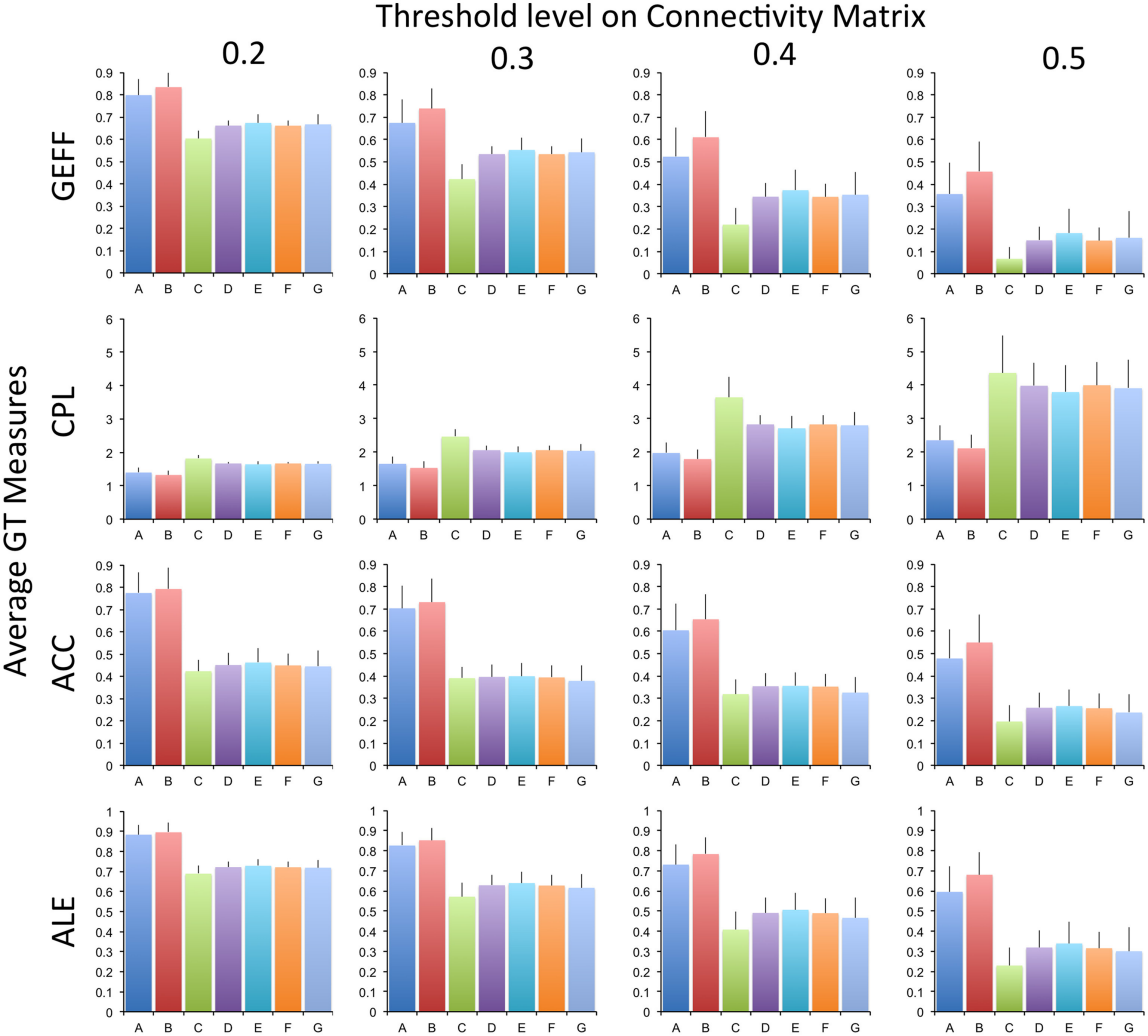
**Graph Theoretical Measurements for each of the preprocessing strategies and threshold level applied to the connectivity matrix**. Error bars indicate the standard deviation across subjects. Graph Theoretical Measurements are GEFF, Global Efficiency; CLP, Characteristic Path Length; ACC, Average Cluster Coefficient; ALE, Average Local Efficiency. Preprocessing strategies (A, B, C, …) are described in Table 2.

Figure 2 shows the average GT measurements for all the preprocessing methods and different thresholds tested. It is evident that preprocessing strategies “A” and “B” differ the most from the other methods. These two pipelines are the ones that employ the least amount of processing steps. Additionally, the average correlation scores within the CM are the largest for methods “A” and “B,” with *r* = 0.2640 and *r* = 0.2967, respectively. As seen in Table 1, similar preprocessing strategies where employed in earlier GT studies. In general, methods “D” through “G” do not differ as much and are typically, in the more recent studied, the preprocessing strategies mostly used. Preprocessing method “C,” which does not include bandpass filtering, is also different in most GT measurements (Table 3) compared to other methods.

**Figure 3:**
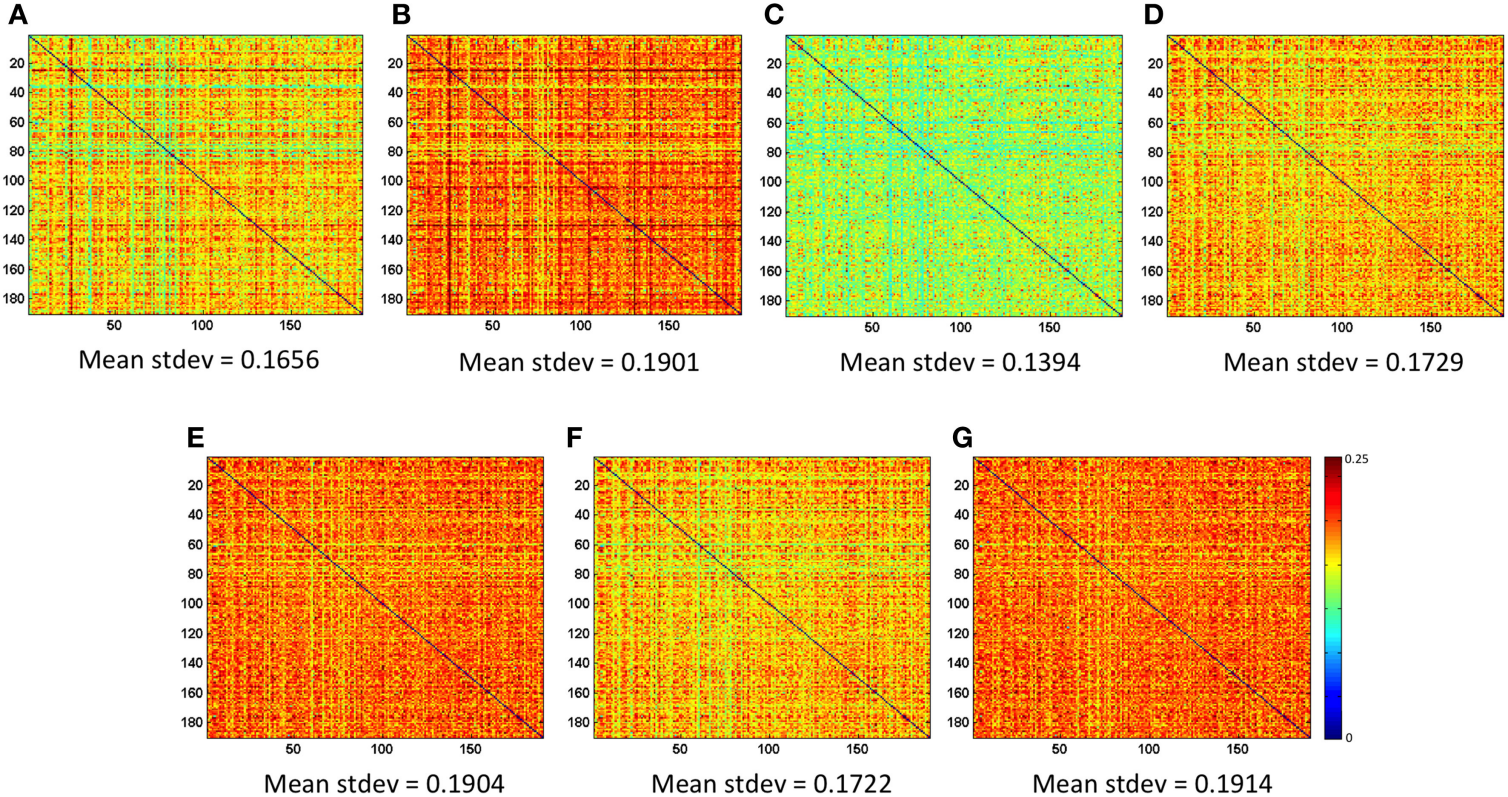
**Standard Deviation Matrices (SDM), which are results from Test 2**. The mean of each SDM is indicated for each preprocessing strategy and is calculated only from the right-superior diagonal of the matrix. Preprocessing strategies (A, B, C, …) are described in Table 2.

Test number 2 had the purpose of evaluating directly the CM across subjects, without calculating the final GT measurements. Reliability was assessed by calculating, at each node pair, the variance in correlation score across subjects within a preprocessing strategy. Figure 3 indicates that, given the preprocessing strategy chosen, there can be an increase or decrease in the variation of the CM across subjects. The lowest variation can be seen in methods “C” (mean stdev = 0.1394) followed by a higher variation seen in method “A” (mean stdev = 0.1656). Methods “D” and “F” have almost identical variation in the CM across subjects, with an average standard deviation of the CM equal to 0.1729 and 0.1722, respectively. The highest average standard deviations are seen in methods “B,” “E,” and “G.”

Test number 3 was performed to verify the relationship of GT measurements and subject head motion. Across the different GT measurements and threshold levels, on average, preprocessing strategy “F” has the lowest correlation with head motion, closely followed by method “D” (Table 4). The measurements of GEFF showed weak to strong correlation with head motion across the preprocessing strategies. Strategies “D” and “F” (in GEFF) showed the lowest amount of correlation with motion in all thresholding levels, with an average correlation of 0.254 and 0.216, respectively. While preprocessing strategy “G” exhibited the highest correlation with motion (mean correlation = 0.706). CPL, ACC, and ALE all demonstrated a similar pattern of results. What is surprising, is that in preprocessing strategy “E” which censors out time points with high motion, does not reduce the dependency of GT metrics on motion. It actually increases the correlation with motion when compared with strategy “D,” which does not contain the censoring step in the preprocessing strategy. Yan et al. (2013b) found similar results to ours regarding head motion. They compared preprocessing using censoring by motion parameters with global signal nuisance regression, and found that using a combination of both increases the relationship of GT measurements and motion estimation, compared to only using global regression. In this paper, we did not perform a preprocessing using only strategy “D” plus global signal regression; therefore, we cannot thoroughly compare our results.

Also, in regards to global signal regression, Liang et al. (2012) investigated the reliability of GT measurements on rs-fMRI and found that the use of the global signal regression produces less reliable results compared to when not using this pre-processing step. This was measured by comparing scans acquired with short and long time intervals. With global signal regression, the average CM among subjects for the “G” preprocessing strategy is close to zero, which may indicate that the number of positive and negative correlation scores are balanced. This corroborates with the study by Braun et al. (2011), where they assessed the reliability of CM and found that the number of negative correlations varied greatly between the different strategies. With the use of the global signal regression, approximately half of the correlations were negative, whereas with the other methods, that number ranges from 0 to 34.8% of the correlation pairs.

In regards to the number of time points removed in strategies “E” and “F,” censoring based on head motion (mean = 6.25 ± 9.75) removed considerably more images when compared to censoring based on outliers (mean = 1.07 ± 4.26). The low number of points that were censored by strategy “F” reveals the reason why strategies “D” and “F” have similar results, exhibited in Table 3. It is also preferable to remove the least amount o time points from your data. It is possible, in a few cases, to remove motion artifact in some images by the use of image processing algorithms. Therefore, there is no need to remove these time points from the data analysis since these images have been corrected. A paper has recently been published that addresses this issue (Power et al., 2014).

This manuscript is limited in the sense that different threshold levels of censoring based on motion and outliers were not evaluated. By modifying the threshold levels it is expected that the end results should change considerably.

Preprocessing strategies A and B have shown similar mean values in all GT measurements (Figure 2). However, the outcomes of using these two preprocessing steps differ significantly from the other five strategies being evaluated, including GT measurements and average correlation score within the connectivity matrix. It is important to emphasize that in the studies by Sanz-Arigita et al. (2010), Van den Heuvel et al. (2008) and Van den Heuvel et al. (2009), they used only a high-pass or band-pass filter. Based on our results, we have shown that it is important to verify which pre-processing strategy is being used, in order to compare GT measurements across different studies that evaluate a similar population. Considering all the tests performed, preprocessing strategies “D” and “F” portray as being the most reliable methods. The only difference between them is that preprocessing strategy “F” includes a censoring based on outliers step. Additionally, they also present results that cannot be statistically differentiated (Table 3). Preprocessing method “D” is a well-established preprocessing pipeline that has been proven to be a reliable scheme (Weissenbacher et al., 2009). There is however a decrease in dependency between on GT measures and head motion when using the censoring based on outliers step (method “F”). Therefore, based on the results, we recommend using preprocessing strategy “F” to increase the reliability across subjects and reduce the dependency on head motion. We were unable to find in the current literature articles that have used censoring based on outliers on GT measures and compared the final outcomes.

We have shown a preprocessing strategy that is reliable among a large homogeneous group, where we point to a method that best reduces the variance across subjects (healthy controls) and controls for head motion. However, in research studies were it is intended to compare clinical populations (i.e., patients vs. controls) a reduction in the variation of the CM might not be optimal. This is the major limitation of this study. Also, different preprocessing strategies can be used and/or developed that were not tested in this study. Finally, further research needs to be performed to establish which preprocessing strategy reduces intra-group variation, while increasing or maintaining inter-group differences.

### Conflict of interest statement

The authors declare that the research was conducted in the absence of any commercial or financial relationships that could be construed as a potential conflict of interest.

## Footnotes

^1^If the noise is Gaussian, then the MAD = σ (2/π)^1/2^; A standard Gaussian N(0, 1) will exceed Q^−1^(p) with probability p.
