# Supplementary material for: Evaluating the reliability of different preprocessing steps to estimate graph theoretical measures in resting state fMRI data
Source: Front Neurosci. 2015 Feb 19;9:48. doi: 10.3389/fnins.2015.00048 (PMC4333797; doi:10.3389/fnins.2015.00048)
Supplement: Supplementary file 1 [file DataSheet1.DOCX]

**Follow-up paired t-tests**

Only indicating where there are no significant statistical different (p>0.05) between the preprocessing methods.

| **GEFF**  **Threshold = 0.2** | **A** | **B** | **C** | **D** | **E** | **F** | **G** |
| --- | --- | --- | --- | --- | --- | --- | --- |
| **A** | - |  |  |  |  |  |  |
| **B** |  | - |  |  |  |  |  |
| **C** |  |  | - |  |  |  |  |
| **D** |  |  |  | - |  | X | X |
| **E** |  |  |  |  | - |  |  |
| **F** |  |  |  |  |  | - | X |
| **G** |  |  |  |  |  |  | - |

| **CPL**  **Threshold = 0.2** | **A** | **B** | **C** | **D** | **E** | **F** | **G** |
| --- | --- | --- | --- | --- | --- | --- | --- |
| **A** | - |  |  |  |  |  |  |
| **B** |  | - |  |  |  |  |  |
| **C** |  |  | - |  |  |  |  |
| **D** |  |  |  | - |  | X | X |
| **E** |  |  |  |  | - |  |  |
| **F** |  |  |  |  |  | - | X |
| **G** |  |  |  |  |  |  | - |

| **ACC**  **Threshold = 0.2** | **A** | **B** | **C** | **D** | **E** | **F** | **G** |
| --- | --- | --- | --- | --- | --- | --- | --- |
| **A** | - |  |  |  |  |  |  |
| **B** |  | - |  |  |  |  |  |
| **C** |  |  | - |  |  |  | X |
| **D** |  |  |  | - | X | X | X |
| **E** |  |  |  |  | - | X |  |
| **F** |  |  |  |  |  | - | X |
| **G** |  |  |  |  |  |  | - |

| **ALE**  **Threshold = 0.2** | **A** | **B** | **C** | **D** | **E** | **F** | **G** |
| --- | --- | --- | --- | --- | --- | --- | --- |
| **A** | - |  |  |  |  |  |  |
| **B** |  | - |  |  |  |  |  |
| **C** |  |  | - |  |  |  |  |
| **D** |  |  |  | - | X | X | X |
| **E** |  |  |  |  | - | X |  |
| **F** |  |  |  |  |  | - | X |
| **G** |  |  |  |  |  |  | - |

| **GEFF**  **Threshold = 0.3** | **A** | **B** | **C** | **D** | **E** | **F** | **G** |
| --- | --- | --- | --- | --- | --- | --- | --- |
| **A** | - |  |  |  |  |  |  |
| **B** |  | - |  |  |  |  |  |
| **C** |  |  | - |  |  |  |  |
| **D** |  |  |  | - |  | X | X |
| **E** |  |  |  |  | - |  |  |
| **F** |  |  |  |  |  | - | X |
| **G** |  |  |  |  |  |  | - |

| **CPL**  **Threshold = 0.3** | **A** | **B** | **C** | **D** | **E** | **F** | **G** |
| --- | --- | --- | --- | --- | --- | --- | --- |
| **A** | - |  |  |  |  |  |  |
| **B** |  | - |  |  |  |  |  |
| **C** |  |  | - |  |  |  |  |
| **E** |  |  |  | - |  | X | X |
| **F** |  |  |  |  | - |  |  |
| **G** |  |  |  |  |  | - | X |
| **H** |  |  |  |  |  |  | - |

| **ACC**  **Threshold = 0.3** | **A** | **B** | **C** | **D** | **E** | **F** | **G** |
| --- | --- | --- | --- | --- | --- | --- | --- |
| **A** | - |  |  |  |  |  |  |
| **B** |  | - |  |  |  |  |  |
| **C** |  |  | - | X | X | X | X |
| **D** |  |  |  | - | X | X | X |
| **E** |  |  |  |  | - | X |  |
| **F** |  |  |  |  |  | - | X |
| **G** |  |  |  |  |  |  | - |

| **ALE**  **Threshold = 0.3** | A | B | C | D | E | F | G |
| --- | --- | --- | --- | --- | --- | --- | --- |
| A | - |  |  |  |  |  |  |
| B |  | - |  |  |  |  |  |
| C |  |  | - |  |  |  |  |
| D |  |  |  | - | X | X | X |
| E |  |  |  |  | - |  |  |
| F |  |  |  |  |  | - | X |
| G |  |  |  |  |  |  | - |

| **GEFF**  **Threshold = 0.4** | **A** | **B** | **C** | **D** | **E** | **F** | **G** |
| --- | --- | --- | --- | --- | --- | --- | --- |
| **A** | - |  |  |  |  |  |  |
| **B** |  | - |  |  |  |  |  |
| **C** |  |  | - |  |  |  |  |
| **D** |  |  |  | - |  | X | X |
| **E** |  |  |  |  | - |  |  |
| **F** |  |  |  |  |  | - | X |
| **G** |  |  |  |  |  |  | - |

| **CPL**  **Threshold = 0.4** | **A** | **B** | **C** | **D** | **E** | **F** | **G** |
| --- | --- | --- | --- | --- | --- | --- | --- |
| **A** | - |  |  |  |  |  |  |
| **B** |  | - |  |  |  |  |  |
| **C** |  |  | - |  |  |  |  |
| **D** |  |  |  | - |  | X | X |
| **E** |  |  |  |  | - |  |  |
| **F** |  |  |  |  |  | - | X |
| **G** |  |  |  |  |  |  | - |

| **ACC**  **Threshold = 0.4** | **A** | **B** | **C** | **D** | **E** | **F** | **G** |
| --- | --- | --- | --- | --- | --- | --- | --- |
| **A** | - |  |  |  |  |  |  |
| **B** |  | - |  |  |  |  |  |
| **C** |  |  | - |  |  |  | X |
| **D** |  |  |  | - | X | X |  |
| **E** |  |  |  |  | - | X |  |
| **F** |  |  |  |  |  | - |  |
| **G** |  |  |  |  |  |  | - |

| **ALE**  **Threshold = 0.4** | A | B | C | D | E | F | G |
| --- | --- | --- | --- | --- | --- | --- | --- |
| A | - |  |  |  |  |  |  |
| B |  | - |  |  |  |  |  |
| C |  |  | - |  |  |  |  |
| D |  |  |  | - | X | X |  |
| E |  |  |  |  | - | X |  |
| F |  |  |  |  |  | - | X |
| G |  |  |  |  |  |  | - |

| **GEFF**  **Threshold = 0.5** | A | B | C | D | E | F | G |
| --- | --- | --- | --- | --- | --- | --- | --- |
| A | - |  |  |  |  |  |  |
| B |  | - |  |  |  |  |  |
| C |  |  | - |  |  |  |  |
| D |  |  |  | - |  | X | X |
| E |  |  |  |  | - |  |  |
| F |  |  |  |  |  | - | X |
| G |  |  |  |  |  |  | - |

| **CPL**  **Threshold = 0.5** | A | B | C | D | E | F | G |
| --- | --- | --- | --- | --- | --- | --- | --- |
| A | - |  |  |  |  |  |  |
| B |  | - |  |  |  |  |  |
| C |  |  | - | X |  | X |  |
| D |  |  |  | - |  | X | X |
| E |  |  |  |  | - |  | X |
| F |  |  |  |  |  | - | X |
| G |  |  |  |  |  |  | - |

| **ACC**  **Threshold = 0.5** | A | B | C | D | E | F | G |
| --- | --- | --- | --- | --- | --- | --- | --- |
| A | - |  |  |  |  |  |  |
| B |  | - |  |  |  |  |  |
| C |  |  | - |  |  |  |  |
| D |  |  |  | - | X | X | X |
| E |  |  |  |  | - | X |  |
| F |  |  |  |  |  | - | X |
| G |  |  |  |  |  |  | - |

| **ALE**  **Threshold = 0.5** | A | B | C | D | E | F | G |
| --- | --- | --- | --- | --- | --- | --- | --- |
| A | - |  |  |  |  |  |  |
| B |  | - |  |  |  |  |  |
| C |  |  | - |  |  |  |  |
| D |  |  |  | - | X | X | X |
| E |  |  |  |  | - | X |  |
| F |  |  |  |  |  | - | X |
| G |  |  |  |  |  |  | - |
